# Inflammation caused by peripheral immune cells across into injured mouse blood brain barrier can worsen postoperative cognitive dysfunction induced by isoflurane

**DOI:** 10.1186/s12860-018-0172-1

**Published:** 2018-09-29

**Authors:** Honghua Zhu, Wei Liu, Hao Fang

**Affiliations:** 1 Department of Anesthesiology, Jinshan Hospital, Fudan University, Shanghai, People’s Republic of China; 2 Department of Anesthesiology, Zhongshan Hospital, Fudan University, No. 180 Fenglin Road, Xuhui District, Shanghai, 200032 People’s Republic of China

**Keywords:** Blood brain barrier, POCD, Inflammation, Immune

## Abstract

**Background:**

Disruption to the blood brain barrier (BBB) is a leading factor associated with the development of postoperative cognitive dysfunction (POCD). Despite this, the underlying mechanism by which BBB disruption promotes POCD in the elderly population has not yet been not fully elucidated.

**Results:**

In this study, we established a POCD mice model using isoflurane, and observed the highly expressed occludin and claudin 5 in brain tissues concomitant with the increased enrichment of CD4 positive cells and NK cells in the hippocampus of POCD mice compared to normal and non-POCD control.

**Conclusions:**

Our data suggests that peripheral immune cells may participate in the inflammatory reaction within the hippocampus, following the administration of anesthesia via inhalation with the destruction of the blood-brain barrier.

## Background

Postoperative cognitive dysfunction (POCD) frequently occurs in the elderly population, 65 years or older following surgery and anesthesia. The hallmarks of POCD include cognitive impairments related to attention, short-term memory, social integration and language comprehension [1–3]. Furthermore, POCD has been reported to diminish quality of life, prolong the hospital stay of patients, and negatively impact the recovery process [4]. Together, these findings reveal a major public health issue, however, the underlying mechanisms are not known.

The blood brain barrier (BBB) acts as a key elements to protect the brain from immune-mediated injuries. This highly specialized endothelium membrane forms a tight barrier, which limits the entry of harmful molecules and immune cells into the brain [5]. In doing so, this creates a unique microenvironment that strictly governs the number of immune cells and immune reactions in the brain [6]. Nevertheless, BBB disruption due to surgery and/or anesthesia may induce the reaction of microglial and astrocyte cells, and lead to neuron impairment.

Neuroinflammation has been identified as an important process for the occurrence and development of POCD [7, 8]. Previous studies have demonstrated that bone marrow-derived macrophages infiltrate the brain [9, 10], whereas microglial activation [11, 12] has been increasingly recognized to contribute to neuroinflammation. Despite this, currently there is a lack of understanding regarding the effects of other peripheral immune cells in the process of neuroinflammation. Peripheral immune cells such as T and B lymphocytes have been reported to play an important role in the inflammatory responses in the Alzheimer’s disease (AD) [13]. In in the hippocampus, reports indicate that the expression of cluster of differentiation (CD) 3+ [14] and CD4+ increase [15] but not CD8+ [16, 17] regulatory T-cells associated AD compared to control. In other words, throughout the development of AD, T-cells may be activated and infiltrate the brain. Similarly, the involvement of natural killer (NK) cells in neurodegenerative diseases such as multiple sclerosis have also been well studied [18]. In AD patients, NK cell activity is decreased [19], which suggests that NK cells may also contribute to the immunopathogenesis of AD. However, it is still unclear the roles in which immune cells play in the brain regarding POCD.

In this study, we investigated the effects of the peripheral immune cells in the process of POCD. This was achieved through the use of POCD mice, and isoflurane was utilized to assess the characteristics of the BBB between POCD and non-POCD mice.

## Methods

### Animal study

C57BL/6 (18 months old) male mice were purchased from Shanghai SLAC Laboratory Animal Co., Ltd. The mice were kept under a 12-hour light/dark cycle in cages contained within a laminar flow system to maintain a pathogen-free environment. The studies were conducted in accordance with the Animal Component of Research Protocol guidelines at Fudan University. The mice were randomly assigned to three groups, which included control, surgery or isoflurane inhalation.: (1) In the control group (*n* = 20), animals received no intervention; (2) in the surgery group (n = 20), animal underwent an appendicectomy under general anesthesia, with 0.5% pentobarbital 0.2 ml intraperitoneal injection. During the appendicectomy, the epityphlon was exposed through a 1–2 cm midline abdominal incision and excised. The wound was then closed by sterile suture. (3) In the isoflurane inhalation group (*n* = 180), mice were placed in a transparent chamber connected with a vaporizer, the dimensions were 30 cm length, 15 cm width and 15 cm height. In each group, mice were kept with 1%, 2% and 3% isoflurane for 2, 4, 6 h respectively, which was followed by a treatment of pure oxygen and mice were woken naturally for the next experiment.

The Morris water maze [20] and open field test [21] were used to evaluate spatial learning, in rodents this is dependent on the hippocampus. Mice were located for escape onto a hidden platform under the water surface in a circular tank (80 cm diameter), which is filled with opaque water (22 ± 1 °C), mice were trained for 5 days (four trails a day). The start position was completely randomized across trails for each individual, while the hidden platform was maintained in the same quadrant for all trails across all training sessions. A 60 s escape latency was noted when mice failed to reach the platform within 60 s. A spatial probe trail was performed on the sixth day after 5 days of training. Escape latency to the platform was recorded by a video camera mounted to the ceiling, and digital images were analyzed by water maze software (HVS Image, UK). For the open field test, in the postoperative sixth day, mice were placed at a columnar vessel (35 cm inner / 80 cm outer diameter and 30 cm height) with 65 dB noise and weak light background for 10 min. The basic steps, periphery distance and central time were all analyzed for anxiety and fear assessment.

Following the tests above, all mice were sacrificed using spinal dislocation and the brain tissue was isolated.

### Flow cytometry

The hippocampus were isolated and digested with 0.1 mg/ml collagenase IV at 37 °C for 20 min and gently passed through a 70 μm strainer. Following this, samples were washed twice with PBS and re-suspended within 5 ml 70% Percoll (GE healthcare, Sweden), then gently treated with 37% Percoll, and centrifuge at 500 g for 30 min. The cell layer between 37 and 70% Percoll were harvested and incubated with the appropriate antibodies (1:50) on ice for 15 min in the dark. Peripheral blood harvested by eyes were used as a positive control and anticoagulated by using 1000 U/ml heparin sodium, which was centrifuged at 13000 rpm for 5 min for the leukocyte harvest. Flow cytometry samples were all stained with 1 μl of 50 μg/ml propidium iode (PI) (Sigma) for 5 min. The cell count was calculated and analyzed by flowjo. All antibodies were purchased from BD Pharmingen or eBioscience: CD3 (FITC, 11–0031-82, eBioscience), CD4 (PE, 12–0041-82, eBioscience), CD8 (PE, 553033, BD), B220 (PE, 562290, BD) and NK1.1 (PE, 17–5941-82, eBioscience). All PI negative cells were defined by the following cell types: NK cells (CD3 − NK1.1+), T cells (CD3+), CD4 T cells (CD3 + CD4+), CD8 T cells (CD3 + CD8+), and B cells (CD3 − B220+).

### Evans blue test

Hippocampus tissue samples of equal weight were immersed in 1 ml of formamide for 72 h at 37 °C. The supernatant was centrifuged at 420,000 g for 20 min and measured respective to the absorbance 632 nm (BioTek, USA). The content of dye was valued to investigate BBB permeability.

### Western blot

Hippocampus tissue were homogenized in RIPA buffer solution and then centrifuged at 4 °C at 13,000 rpm for 10 min. The protein quantity in the supernatant was determined using a BCA protein assay kit (Well-bio, China). Equal amounts of protein samples were separated by sodium dodecyl sulfate–polyacrylamide gel electrophoresis (SDS–PAGE) and transferred to polyvinylidene fluoride membranes. The membranes were then blocked in 5% skim milk TBS for 90 min and then incubated with the respective primary antibodies overnight at 4 °C: rabbit anti-occludin polyclonal (1:1000, Invitrogen), and mouse anti-claudin-5 monoclonal (1:2000, Invitrogen). Membranes were washed in TBST and incubated with goat anti-mouse (1:5000, Invitrogen) and goat anti-rabbit (1:5000, Invitrogen) secondary antibodies at room temperature (RT) for 1 h. Membranes were then treated with an enhanced chemiluminescence detection kit (Millipore), and the intensity of each band was quantified by densitometry. Relative expression was normalized to that of β-actin (1:10000; Abcam).

### Immunofluorescence assay

Hippocampus tissue samples were isolated and gently washed with PBS, and fixed with 1% paraformaldehyde in RT for 10 min. Following this, samples were quenched using 0.125 M glycine then permeabilized with 0.1% Triton X-100 and blocked with 5% BSA for 30 min at 37 °C. Subsequently, samples were incubated with Occludin and Claudin 5 antibodies at 4 °C overnight. After washing, cells were further incubated with the appropriate Alexa Fluor secondary antibody at 1:20000 dilutions for 30 min at RT. After washing, cells were mounted in mounting media with DAPI (Vector Laboratories, Burlingame, CA). The expression and localization of Occludin and Claudin 5 were digitally captured at 400X magnification and acquired using Image J software.

### Data analysis

Data calculation and statistical analysis was performed using Graphpad Prism 5.0. Comparisons amongst multiple groups were analyzed using Student’s t test. *p* values less than 0.05 were considered as statistical significance.

## Results

### Identification of POCD model treated with isoflurane

**Fig. 1:**
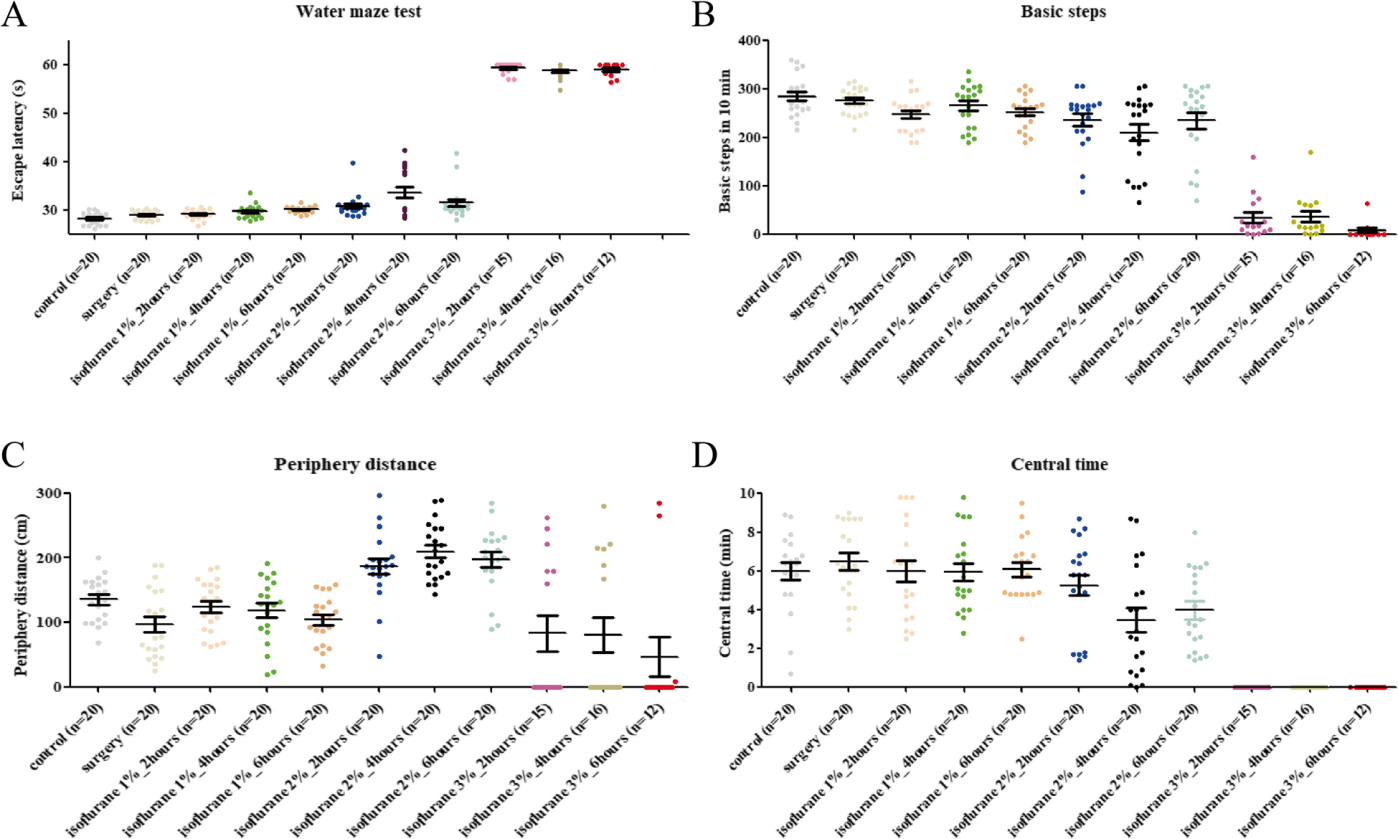
POCD mice identified using water maze and open field test. The indexes of escape latency period (**a**) of water maze as well as basic steps (**b**), periphery distance (**c**) and central time (**d**) of open field test. “*” represents statistical significance

Initially, to optimize the efficiency while establishing the POCD model, mice were treated at different doses and for durations of isoflurane. We observed that the groups of mice treated with 3% isoflurane for 2, 4 and 6 h died in 5, 4 and 8 in total 20 mice respectively, while no death occurred in mice treated with 1% and 2% isoflurane. In addition, we noted that the surviving mice treated with 3% isoflurane displayed extreme impairment to their nervous system compared to POCD mice. These mice failed the water maze or open field tests (Fig. 1). Furthermore, differences in the water maze test in mice treated with 1% isoflurane were non-significance for both control and surgery groups (Fig. 1a). In mice treated with 2% isoflurane, we found that the rate of mice displaying an obvious loss of memory (40%) were treated with isoflurane for more than 2 h (5%) and 6 h (10%). Overall, the escape latency period was higher than control group (2 h, *p* = 0.041; 4 h, *p* = 0.027; 6 h, *p* = 0.038) (Fig. 1a). These 11 mice (1 in 2 h, 2 in 6 h and 8 in 4 h) were categorized with POCD identified by the water maze test. Likewise, the open field test was conducted to further validate the POCD model (Fig. 1b-d). Most of the mice treated with 3% isoflurane were not able to move in the vessel. Following this, we observed that the groups of mice treated with 2% isoflurane for 4 h had the greatest response to fear and anxiety compared to other groups (2 in 2 h, 4 in 6 h and 7 in 4 h) according to the indexes of basic steps, periphery distance and central time. Taken together, we concluded that 2% isoflurane treatment for 4 h may be an optimal condition for POCD mice model preparation. Subsequently, we harvested the 9 POCD mice and the other 51 non-POCD mice from 2% isoflurane identified by behavioral experiments.

### BBB disruption in the POCD model

**Fig. 2:**
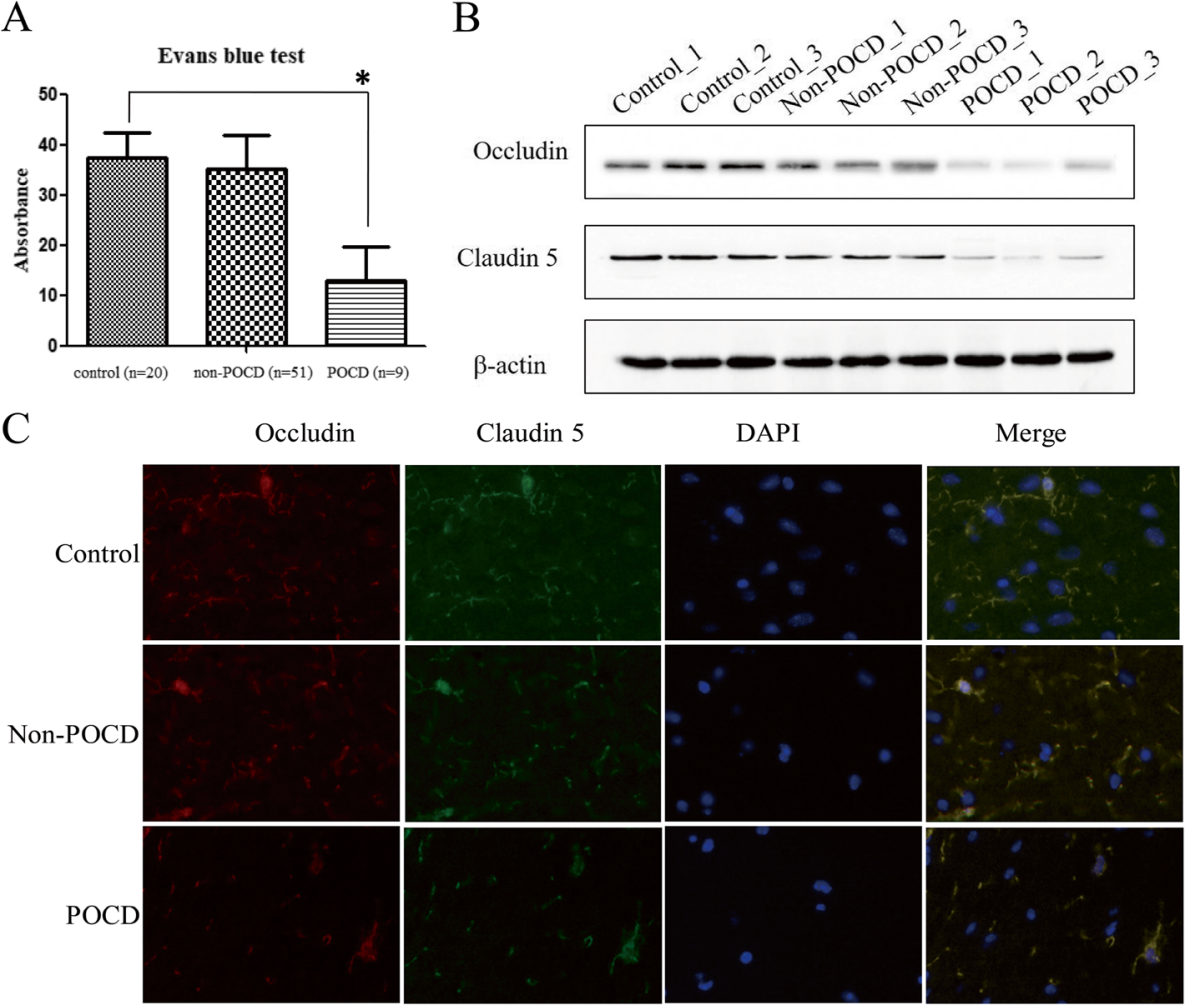
BBB situation of POCD. The absorbance of evans blue (**a**) and Occludin and Claudin 5 expression in hippocampus by WB (**b**) and IF assay (**c**) in POCD. “*” represents statistical significance

Next, we investigated the permeability of the BBB in POCD and non-POCD mice. We observed that the absorbance of evans blue in the supernatant of POCD mice was lower compared to control and non-POCD groups, which indicated that the missed part of the dye could permeate into brain tissues through BBB in POCD group but not in control and non-POCD group (Fig. 2a). Consistently, the expression of Occludin and Claudin 5 was observed to be reduced in the hippocampus of POCD mice compared to control and the non-POCD group in all three individual mice. This was determined by WB and IF (Fig. 2b, c).

### Infiltration of peripheral CD4+ T and NK cells through BBB in POCD

**Fig. 3:**
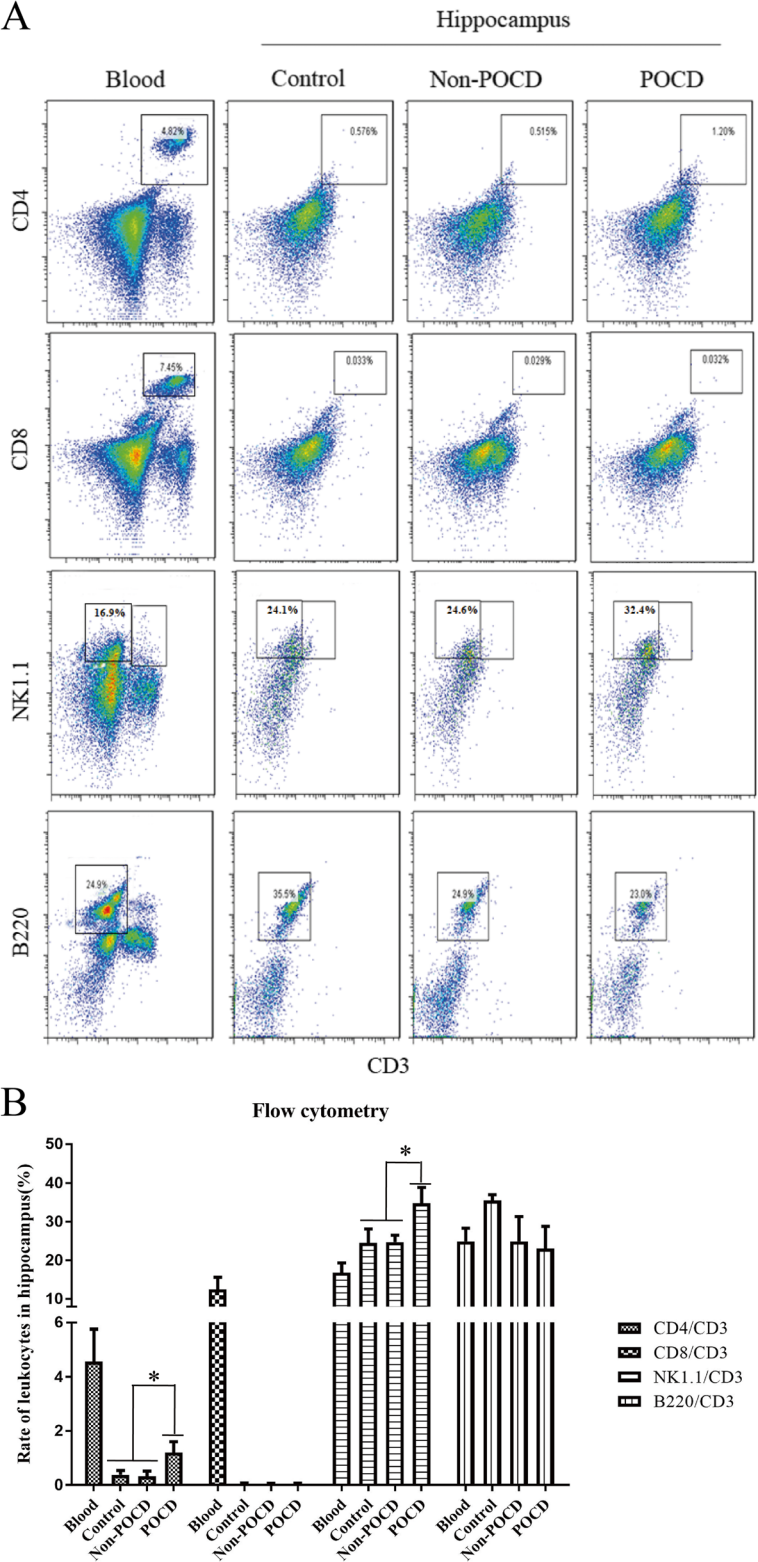
Peripheral leukocytes in hippocampus of POCD calculated using flow cytometry. Flow cytometry sorting (**a**) and statistic analysis (**b**) of leukocytes in hippocampus. “*” represents statistical significance

Next, we investigated the role of immune cells including T, B and NK cells from peripheral blood in the brain tissues, taken from individual mice (*n* = 5). Here we focused on the hippocampus, which is responsible for temporary memory and emotion building. Using leukocyte biomarkers we detected peripheral immune cells in hippocampus by flow cytometry (Fig. 3a). Furthermore, we observed that CD4+ T and NK cell expression was higher in the hippocampus of the POCD group compared to the control and non-POCD group. Conversely, no significant differences were detected in CD8+ T and B cells (Fig. 3b). Collectively, this suggests that CD4+ T cells and NK cells from peripheral leukocytes infiltrated past the BBB in POCD mice.

## Discussion

Postoperative cognitive dysfunction (POCD) is recognized as a complication associated with surgery and anesthesia. Although a number of perioperative factors have been implicated, the pathogenic mechanisms of POCD remain largely unknown. According to previous studies [22–25], surgery activates the host’s innate immune system resulting in peripheral inflammation. This is characterized by elevated levels of proinflammatory cytokines, however, current evidence to suggest that the central nervous system (CNS) is directly damaged by inhalation anesthesia or surgical procedures lacking. Therefore, this raises the question regarding the source of these inflammatory factors. Recent studies revealed that the expression of inflammatory cytokines may be caused by the activation of microglia cells in the hippocampus [11, 12]. Meanwhile, it has also been suggested that the overexpression of inflammatory cytokines may be due to the infiltration of bone marrow-derived macrophages into the hippocampus [9, 10]. Thus, our study provides evidence that cognitive impairment is associated with enhanced production of T cells and NK cells in the hippocampus following surgery and anesthesia in elderly mice.

Surgery and anesthesia are two major factors in the development of POCD. Previous studies to investigate this phenomenon have primarily focused on general anesthetic drugs. In particular, isoflurane which is known to be an especially volatile anesthetics [2, 22]. In our study, isoflurane was used to establish a model for POCD. In our study, for model preparation, we determined the optimal condition was 2% isoflurane administered for 4 h, which yielded similar results to a surgical operation. The rate of the POCD model establishment reached 35–40% according to water maze or open field test (Fig. 1). Due to ethological functional differences between the water maze and open field tests, intersection of mice from these two experiments was used to determine the mice which fit the POCD model. This was done in order to minimize individual variation.

Cognitive decline may be also attributed to the breakdown of the BBB [26, 27]. The BBB is formed mainly by intercellular tight junctions [28], such as occludin and claudin-5. Here, we found that the expression of both claudin-5 and occludin were decreased in the hippocampus of POCD mice compared to control and non-POCD mice (Fig. 2b, c). This finding, strongly supports the observations which suggests the BBB is damaged in POCD mice, which may lead to abnormal behavioral performance and impaired BBB function. Nevertheless, it is difficult to determine whether the breakdown of the BBB is a primary cause of entry for immune cells or just a consequence of diffusing cerebral inflammatory events. For non-POCD mice, the presence of reduced Claudin 5 and stable occludin expression indicates two points. First, individual difference play an important role in POCD occurrence, and secondly no clear distinctions in the expression of biomarkers have been established between POCD and non-POCD conditions. Here, we speculate that the occurrence of POCD is largely dependent on the cerebral function of individual per se.

The mammalian CNS is an immune privileged site, shielded behind barriers from direct interaction with circulating immune cells. Although, further research is required to address the role of immune cells such as NK, B, and T cells in the pathogenesis of POCD. It is clear that events leading to neuroinflammation are definitely associated with impairment to brain function, inhibition of hippocampal neurogenesis, disruption to cognitive ability, and contribute to illness and depression [29–31]. Therefore, in our study, we detected the distribution of peripheral immune cells in the hippocampus using flow cytometry. Here, we did not observe any differences in B cells as a result of isoflurane treatment (Fig. 3), which suggests that B cells do not contribute to the neuroinflammatory process associated with POCD. In addition, CD4+ but not CD8+ T cells were significantly evaluated in the hippocampus of POCD samples compared to their control and non-POCD counterparts. This suggests that CD4+ T cells may play a role in the proinflammatory process in POCD development. CD4+ T cells in circulating lymphocytes are unique due to their relationship to brain self-antigens, which have been reported to play a pivotal role in supporting brain plasticity. This occurs both at basal and in response to CNS trauma [32, 33]. Moreover, we observed that population of NK cells were higher in the POCD group compared to the control and non-POCD groups analyzed. NK cells involvement has been previously reported in neurodegenerative diseases such as multiple sclerosis and AD [18, 19]. However, it should be noted that there is a possibility that infiltration of NK cells into the BBB is not a defensive reaction but a result of POCD progression, which leads to the activation of immune system.

## Conclusion

Our study determined that peripheral immune cells participate in the immune inflammatory response in the hippocampus following surgery and the inhalation of anesthesia. Using the POCD model we established, our data demonstrates that specific biomarkers can also be associated with the destruction of the BBB. In summary, it is suggested that the peripheral immune cells aggravate the inflammatory response and enhance the impairment of brain tissues in POCD.

## Abbreviations

AD: Alzheimer’s disease
BBB: Blood brain barrier
CD: Cluster of differentiation
NK: Natural killer
POCD: Postoperative cognitive dysfunction
